# Characterization of the complete chloroplast genome of *Salix magnifica*, a vulnerable species endemic to China

**DOI:** 10.1080/23802359.2019.1710602

**Published:** 2020-06-02

**Authors:** Jinfeng Liu, Siyan Liu, Rui Wang, Shuyan Guan, Jing Qu

**Affiliations:** College of Life Sciences, Jilin Agricultural University, Changchun, China

**Keywords:** *Salix magnifica*, vulnerable species, chloroplast genome

## Abstract

*Salix magnifica* is only sporadically distributed in the western part of Sichuan, the area of the willows is narrow. There are not many plants, and they easily fall into the endangered state. It is currently on the International Union for Conservation of Nature (IUCN) red list of threatened species. In this study, we first assembled the complete chloroplast (cp) genome of *Salix magnifica* by Illumina paired-end reads data. The whole genome was 154,977 bp, consisting of a pair of inverted repeats of 27,457 bp, large single-copy region, and a small single-copy region (71,119 and 16,205 bp in length, respectively). The cp genome contained 116 genes, including 80 protein-coding genes and 36 tRNA genes. The overall GC content of the whole genome was 36.7%. A neighbour-joining phylogenetic analysis demonstrated a close relationship between *Salix magnifica* and *Salix oreinoma*.

*Salix magnifica* is the genus of the Salix and the branch of the Willow, named after its large leaves (Wu et al. 2019). It is grown in western part of Sichuan and was found in the mountains at an altitude of 2100–2800 m. *Salix magnifica* is a mesophyte-positive hi-light tree species, which has the characteristics of resistance to cold, wind, fear of flooding and sand burial, and rapid growth (Wang et al. 2014). Both protective forests and mining forests can be developed. It also has certain medicinal value and has antipyretic and analgesic functions. It can also used for symptoms such as jaundice, hemoptysis, vomiting blood, and blood in the stool (Lei et al. 2017). *Salix magnifica* has become a dangerous species, and nature reserve has been established in Sichuan. The distribution of the willows in the protected area is concentrated, so long as the cutting is not carried out and the introduction and propagation are carried out, the effective protection can be obtained.

In this study, *Salix magnifica* was sampled from the Endangered Species Reserve of Jilin Agricultural University Changchun County, China (125°19′E, 43°43′N). A voucher specimen (JF20193514) was deposited in the Herbarium of the Plant Biotechnology Center of Jilin Agricultural University, Changchun, China.

The present study is the first time to assemble and characterize the complete chloroplast genome for *Salix magnifica* (GenBank: NC_037424.1) from hight-hrough-put sequencing data. The existing chloroplast Genome sequence of Salix magnifica was downloaded from the National Center for Biotechnology Information’s Organelle Genome Resources database (NC_037424.1) as the reference sequence, and the chloroplast Genome of Salix magnifica was assembled using SPAdes v3.6.0 software (Bankevich et al. 2012). The default setting of parameters was adopted. Sequence annotation first confirmed the availability and boundary of genes by blastn comparison directly through the protein-coding sequence of the proximal species. Then, the genes in the chloroplast genome were annotated by online tool DOGMA (http://dogma.ccbb.utexas.edu/) with default parameters, and the genes were functionally annotated by combining with NR (http://www.ncbi.nlm.nih.gov/) database (Lohse et al. 2013). TRNA was annotated using the trnascan-se online site. RNAmmer 1.2 Server (HTTP//www.cbs.dtu.dk/services/RNAmmer/) was used for rRNA comments. The chloroplast genome of Salix magnifica was mapped using OGDRAW (HTTP//OGDRAW. Mpimp-golm.mpg. DE/cgi-bin/OGDRAW. Pl) software (Asaf et al. 2017).

The complete cp-DNA of *Salix magnifica* was a circular molecule 154,977 bp in length, comprising a large single-copy (LSC) region of 71,119 bp and a small single-copy(SSC) region of 16,205 bp, separated by two inverted repeat regions (IRs) of27,457bp. It contained 116 genes, including 80 protein-coding genes and 36 tRNA genes.The phylogenetic tree reveals all the species of Willow branch formed a monophyletic clade with high-resolution value and *Salix magnifica* is most related with *Salix oreinoma* (Figure 1).

**Figure 1.**
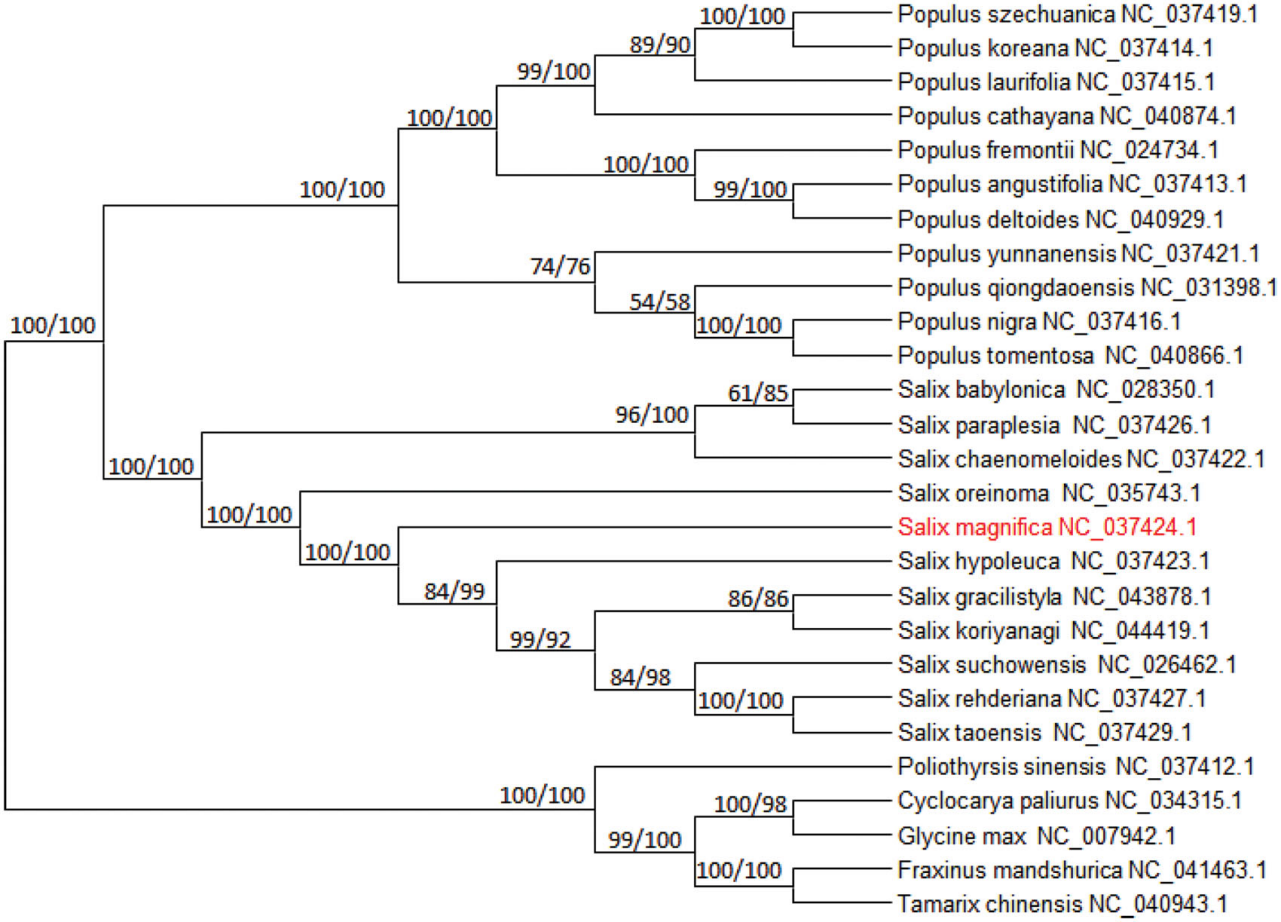
The phylogenetic tree based on 27 complete plastid genome sequences.
